# Conjugated Polymer Process Ontology and Experimental Data Repository for Organic Field-Effect Transistors

**DOI:** 10.1021/acs.chemmater.3c01842

**Published:** 2023-10-25

**Authors:** Aaron L. Liu, Myeongyeon Lee, Rahul Venkatesh, Jessica A. Bonsu, Ron Volkovinsky, J. Carson Meredith, Elsa Reichmanis, Martha A. Grover

**Affiliations:** † School of Chemical & Biomolecular Engineering, Georgia Institute of Technology, 311 Ferst Drive, Atlanta, Georgia 30332, United States; ‡ Department of Chemical & Biomolecular Engineering, Lehigh University, 124 East Morton Street, Bethlehem, Pennsylvania 18015, United States

## Abstract

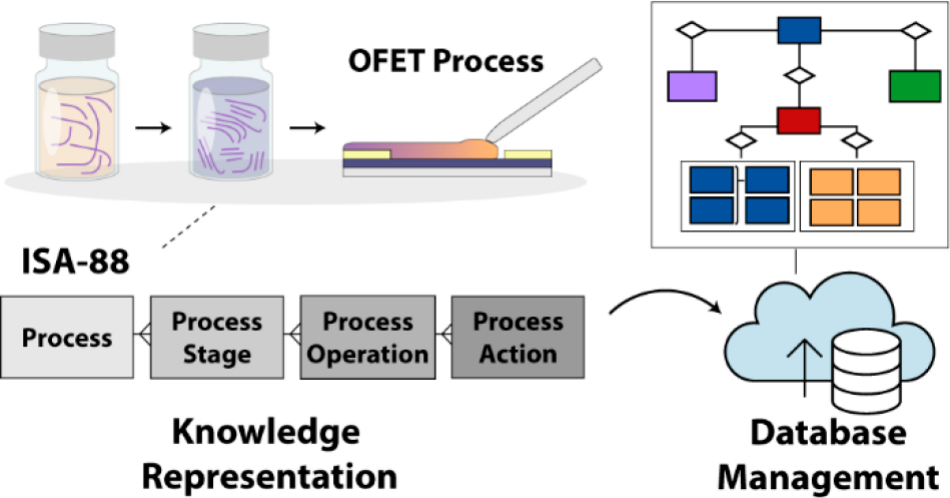

Polymer-based semiconductors and organic electronics encapsulate a significant research thrust for informatics-driven materials development. However, device measurements are described by a complex array of design and parameter choices, many of which are sparsely reported. For example, the mobility of a polymer-based organic field-effect transistor (OFET) may vary by several orders of magnitude for a given polymer as a plethora of parameters related to solution processing, interface design/surface treatment, thin-film deposition, postprocessing, and measurement settings have a profound effect on the value of the final measurement. Incomplete contextual, experimental details hamper the availability of reusable data applicable for data-driven optimization, modeling (e.g., machine learning), and analysis of new organic devices. To curate organic device databases that contain reproducible and findable, accessible, interoperable, and reusable (FAIR) experimental data records, data ontologies that fully describe sample provenance and process history are required. However, standards for generating such process ontologies are not widely adopted for experimental materials domains. In this work, we design and implement an object–relational database for storing experimental records of OFETs. A data structure is generated by drawing on an international standard for batch process control (ISA-88) to facilitate the design. We then mobilize these representative data records, curated from the literature and laboratory experiments, to enable data-driven learning of process–structure–property relationships. The work presented herein opens the door for the broader adoption of data management practices and design standards for both the organic electronics and the wider materials community.

## 1 Introduction

The domain of conjugated polymer semiconductors is of keen interest to both the materials informatics and the organic electronics communities due to the promising opportunities this class of materials offers for large-area, printable, deformable electronic devices and energy applications.^1−3^ In the years since the advent of the Materials Genome Initiative in 2011, conjugated polymer semiconductors have been associated with over 5000 peer-reviewed articles (Web of Science, March 2023).^4^ A subset of this body of literature has striven to further accelerate knowledge discovery in this materials space through applied data science, machine learning, and high-throughput experimentation techniques. For example, data-driven techniques have recently been leveraged to pursue targeted advances for thin-film device applications including organic field-effect transistors (OFETs),^2,5−8^ organic light-emitting diodes (OLEDs),^9−12^ and organic photovoltaics (OPVs).^13−17^ Early successes include the application of self-driven laboratory workflows to screen quaternary OPV formulations at the full device level.^13,18^ Indeed, these endeavors have positioned organic electronics as a significant research thrust within the materials informatics community.

Despite recent accomplishments, rational design of organic electronic devices, particularly those that are polymer based, still largely materializes through one-parameter-at-a-time, hypothesis-driven studies due to the limited availability of representative experimental data. The conjugated polymer materials domain is a research area with a compelling need for experimental data management solutions. While a few examples of shared data sets or databases that target organic electronics research have been reported, such as the Harvard Clean Energy Project^19^ and OCELOT,^20^ they mostly include computational data on small molecules. A recent effort in Deep4Chem mined over 1000 peer-reviewed articles to build an experimental database of chromophores,^21^ but similar to prior efforts it largely targets electronic structure–property measurements and is not inclusive of process history. In extending database management effectively to polymer-based devices, providing data models that are inclusive of experimental processing information are priorities for storing accurate and reproducible records.

Experimental database design and management for polymer electronics however is nontrivial, especially when a plurality of the relevant attributes related to the provenance of the sample must be included accurately to form a robust, “reusable” data record. Data ontologies are not standardized in the organic electronics space: fully capturing all relevant experimental information is challenging, and organic device performance is highly sensitive to the many parameters associated with the active layer deposition process. For example, the charge-carrier mobility (μ), a key figure of merit for OFETs, has been shown to vary significantly for poly(3-hexylthiophene) (P3HT) (∼10^–6^–10^0^ cm^2^/V·s)^8,22^ and poly[2,5-(2-octyldodecyl)-3,6-diketopyrrolopyrrole-*alt*-5,5-(2,5-di(thien-2-yl)thieno[3,2-*b*]thiophene)] (DPP-DTT) (∼10^–5^–10^1^ cm^2^/V·s).^8^ This performance variation is attributed to not only batch-to-batch characteristics of the polymer but also a plethora of parameters related to the polymer’s process history, starting with the solution state through the thin-film deposition process.^23,24^ Another source of variation is that mobility values are derived from device measurements via model fitting, and employing different methods/parameters (i.e., models, measurement settings, voltage limits) may affect the extracted mobility value. Recording processing and measurement parameters provides indispensable contextual value to organic device data, but nonetheless, recording all of them efficiently is not straightforward. Additionally, since the design space is inherently dynamic due to the evolving nature of research, data models must be designed with flexibility in mind without sacrificing consistent vocabulary. Thus, generating a representative data ontology describing the experimental device realm is a challenge that must be addressed to enable reliable database designs.^25^

Though process representations are not new problems for the sake of curating materials databases, navigating these challenges for experimental polymer domains has only been explored recently by a minority of materials data researchers. The experimental database effort in MaterialsMine promotes the inclusion of processing terms for polymer nanocomposites,^26−28^ while the Community Resource for Innovation in Polymer Technology (CRIPT) proposes a framework to comprehensively describe polymer data, seeking to unify all aspects of sample provenance from synthesis, processing, characterization, properties, and instrumentation/citation metadata.^29^ These active endeavors open the door for a broader adoption of polymer-based data management solutions, but it is up to various communities to enable tailored data models for their specific subdomains.^30^

In this work, we used OFETs as a model system to propose an experimental data ontology associated with semiconducting polymer processing. We then produced a data structure that focuses on the deposition of the active semiconducting polymer layer and leveraged it to implement an experimental repository relating the semiconducting polymer process history to device performance. To guide a robust representation of that process history, this work draws upon ISA-88, an international standard for automation in batch process control, to construct generalizable relationships across process transformations within the fabrication procedure to create the semiconducting thin film.^31^ Building a data repository that can handle the many nuances of this complex design space is expected to provide a platform to enhance hypothesis design, scientific decision making, and model development within the traditionally “small data” space of the organic electronics community.

## 2 Data Model and Knowledge Representation

### 2.1 Parameter Space

**Figure 1:**
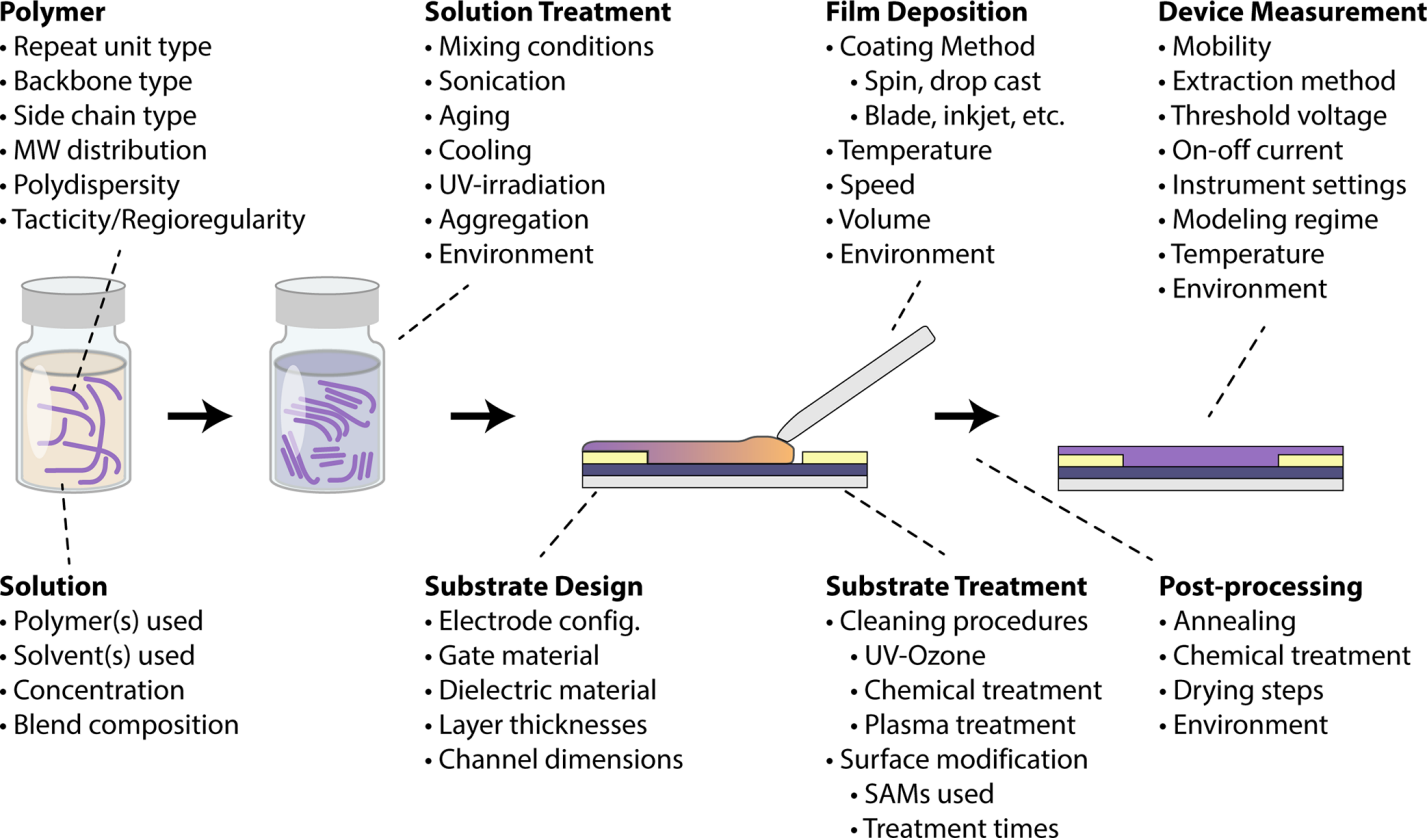
Overview of the process associated with transforming a semiconducting polymer into the active layer of an OFET sample, including the common parameters associated with a sample recipe.

Defining the required information to capture is facilitated by published reporting standards for experimental OFET device data.^32−34^ An overview of the major materials and process stages involved in depositing the semiconducting polymer layer and a nonexhaustive set of their related parameters/attributes is presented in Figure 1. A device recipe considers the starting materials—a polymer and a device substrate—and tracks these two inputs as they are transformed through a series of process steps and ultimately integrated into the output: an OFET on which a device measurement is made. Important parameters and nuances to evaluate device performance include materials characteristics, solution processing, substrate treatment, and the instrument parameters and models used to extract device metrics.

The primary material parameters describe the components of the active semiconducting layer as well as any other materials (i.e., solvents, chemical treatments) included in the processing procedure. The polymer and any other components are dissolved into a solution that is ultimately deposited onto the device substrate. As the behavior of the polymer in the solution state is a key determinant of its thin-film behavior, all information associated with the solution makeup and its processing is especially crucial to capture for data provenance purposes.^35^ Choices in the material characteristics of the polymer (i.e., molecular weight distribution, regioregularity, tacticity, etc.) yield a range of structural and morphological motifs that in turn significantly impact device performance.^36^ The identity of the solvent(s) used affects not only the polymer–solvent interactions in the solution state but also the dynamics of the thin-film deposition process, thereby influencing the structure and performance of the final active layer.^37^

The OFET device substrate is a layered device structure, comprising substrate, gate electrode, dielectric, and source/drain electrodes. The most common electrode configurations used in the organic electronics community include (a) bottom-gate bottom-contact (BGBC), (b) bottom-gate top-contact (BGTC), (c) top-gate bottom-contact (TGBC), and (d) top-gate top-contact (TGTC). The electrode configuration is a necessary contextual detail for reporting a device measurement, as substrate designs often take advantage of charge-carrier behavior at different interfaces. Additionally, as substrate design parameters (i.e., channel dimensions such as width and length)^7^ and material choices (i.e., electrode material, dielectric, etc.) may influence device measurements,^38^ this design information is important to include in a data entry to promote experimental reproducibility.^32^

An emphasis of the work herein is that processing information is indispensable for the purpose of storing reproducible device data. Seemingly minor differences in processing can lead to significant changes in the recorded charge-carrier mobility of an OFET sample. Omitting information related to this process history will therefore lead to errors or inaccuracies when comparing device data across experiments. Even prior to deposition, the solution and device substrate undergo process transformations that can affect the deposited thin-film and device characteristics. Solution-based processes may include operations such as sonication, aging, poor solvent addition, cooling, etc.^39^ in a prescribed sequence to promote solution-state aggregation,^40−42^ while the surface pretreatment procedure may include, for example, a cleaning process (e.g., UV-ozone or plasma treatment) followed by a surface modification step via self-assembled monolayer (SAM).^43^ As an example, differences in solution aging times (i.e., 3, 6, and 24 h prior to coating) can lead to noticeable structural changes that affect the final value of the device measurement in P3HT.^44,45^

The coating process could be performed through a plethora of solution-casting methods including drop casting, spin coating, blade coating, inkjet printing, slot die coating, etc., that all have different physical impacts on thin-film morphology and therefore device performance, especially when coupled with solution pretreatment.^46,47^ Meniscus-guided coating techniques, for example, yield a set of deposition regimes governed by a complex parameter space that includes flow conditions, coating speeds, stage temperatures, drying times, contact angles, etc.^48^ In combination with the solution properties and surface interactions, coating conditions are often chosen carefully to tune the morphology of the deposited thin film. Postprocessing operations may also be performed after the coating stage, such as annealing^49^ or selective etching,^50^ to further control the thin-film morphology. Throughout all processing steps, the ambient environment (humidity, air vs inert atmosphere, temperature, etc.) may also play a role in the final properties.^51^

Instrument settings are classified as attributes of the measurement rather than the fabrication process, but this metadata is important as it contextualizes the reported value. Particularly, OFET performance metrics can be nontrivial to represent because a charge-carrier mobility value is not a measurement per se; it is a parameter value derived from curve fitting of the actual measurement, a transfer curve sweep. Therefore, the measurement and fitting protocol used to extract the charge-carrier mobility benchmark from the actual transfer curve is an important consideration. Unreported details about measurement regimes, voltage sweep range/direction, the measurement environment, etc., can lead to misinterpretations about the provenance of device metrics such as the mobility. In some cases, mobilities extracted from the same device data can differ significantly when different extraction methods are used (e.g., space-charge-limited current–voltage (SCLC), time-of-flight (ToF), etc.)^52^ or when different voltage ranges are chosen to obtain the fitted mobility value.^53^ Guidelines on robust mobility extraction protocols and measurement metadata reporting are relevant here and are elaborated on in the literature.^52−54^

### 2.2 Sample Representation

**Figure 2:**
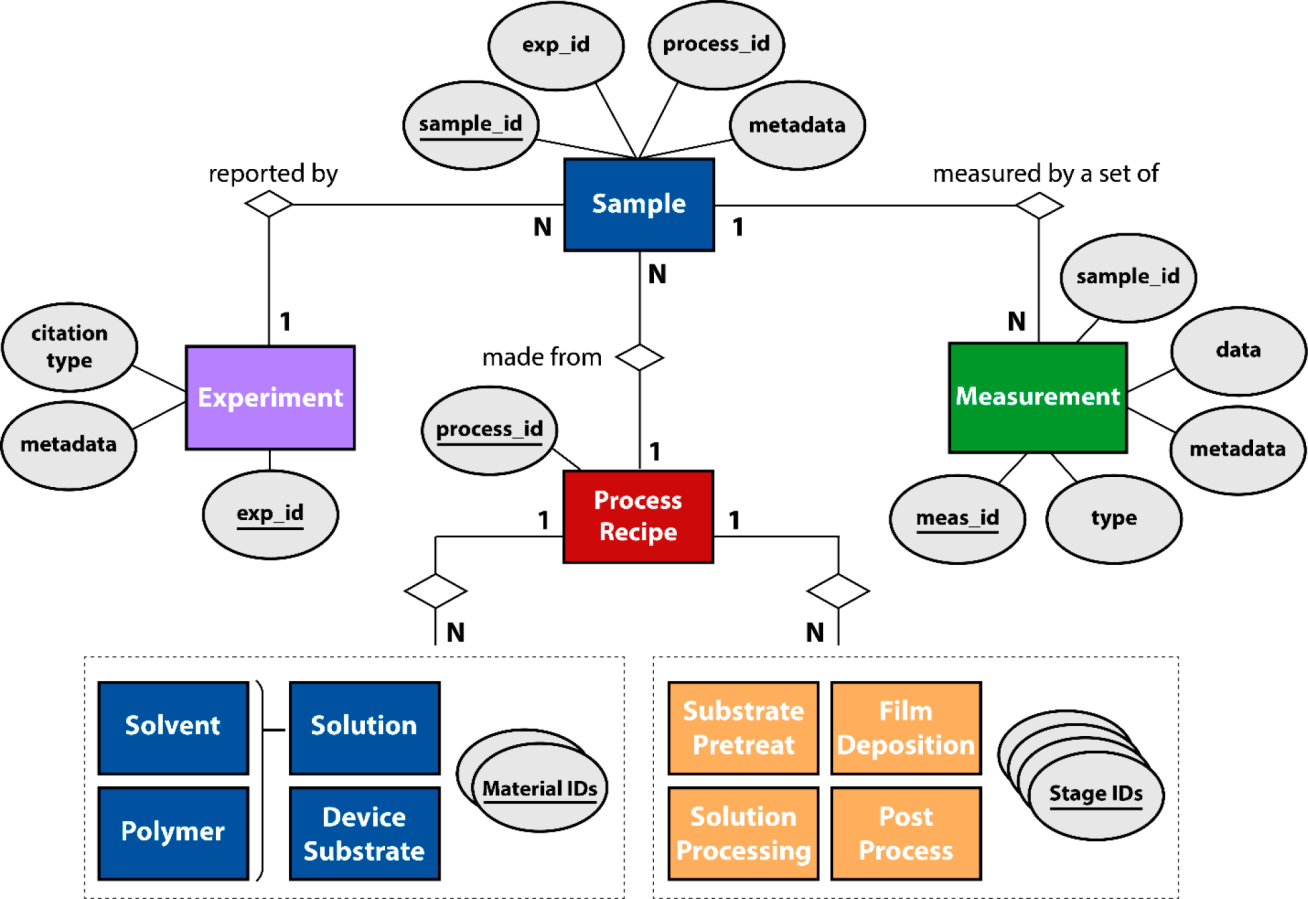
High-level entity–relationship diagram depicting the reporting of an experimental sample, including relationships with experiment information, the process recipe, and the associated measurement data. Gray ovals are attributes associated with the labeled entities. Inputs to the process recipe are represented by material nodes and process stages (see Figure 3).

The translation of the real-world parameter space and its relationship to a robust data model requires definition and elaboration of an ontology. We direct the reader to introductory SQL and database literature to facilitate conceptual understanding of the database model enumerated below.^30,55^ At a high level, the entity–relationship diagram in Figure 2 shows how an organic device sample with its associated reported measurement (i.e., a charge-carrier mobility value) may be conceptually encapsulated as an experimental data record. This diagram provides an important visualization of how various parameters, data, and information in the experimental real world are captured as attributes of related objects to facilitate the organization of data in constructing a database. Rectangles represent entities or objects, diamonds represent relationships between entities, and labeled ovals represent attributes containing the data or information associated with the various entities, where underlined labels denote a unique identifier for that object.

A sample refers to a single organic device in the form in which all of its characterization data (a set of measurement objects) were collected and contain explicit information associating it with its reporting origin (experiment) and its physical origin/process history (process) (Figure 2). Sample has a one-to-many (1–*N)* relationship with measurement, since a single OFET could be measured a number of times by a variety of characterization methods not limited to device performance. A measurement has a type (e.g., transfer curve, spectroscopy, scattering, thickness, etc.) and the heterogeneous data (e.g., value type, value, error, etc.) and metadata (e.g., instrument information, date measured, etc.) associated with it. An experiment refers to the source information associated with the reporting of the sample and therefore has a citation type (e.g., laboratory, journal article, dissertation) and metadata associated with that source (e.g., digital object identifier (DOI), date published, author information, etc.). Experiment and sample have a one-to-many relationship as a single experiment may report multiple samples. A process recipe refers to the unique material ingredients and process sequence through which the sample was generated. The process recipe contains foreign keys (i.e., references to material nodes) that link information about the device substrate and the solution (the latter contains polymer and solvent information) and to process stage nodes that subdivide the process history (vide infra). Metadata fields linked to the material entities contain information such as polymer batch or lot information, supplier information, etc. These material nodes also serve as placeholders to expand the data model to include more details on synthetic routes (for polymer) and/or device fabrication routes (for device substrate) in future database development. Sample and process recipe have a many-to-one (*N*–1) relationship as a given sample device can only be associated with one process recipe but the same process recipe could be used for multiple samples.

### 2.3 Process Representation

Comparing device data reported from multiple sources requires that the various nuances of the experimental design space are accurately represented in a robust data format. Particularly, understanding the sensitivity of the process space is non-negotiable for the sake of reproducibility and accurate data representation. However, in contrast to the other entities in Figure 2, it is not straightforward to manifest a data structure that broadly represents the process history for the conjugated polymer layer. This is not only because the real-world process history is extremely complex but also because the various events in a process history have an explicit order, and the events may not occur consistently across samples. For example, as discussed above, the solution processing procedure may include sequenced pretreatment techniques to induce polymer aggregation in the solution state. This procedure may include multiple steps, and the ordering of those steps may affect the final film and properties (i.e., sonication and then aging or aging and then sonication).^44^ The example above exemplifies a broader challenge in robustly handling information in both dynamic and nuanced ways in sample recipes.

One avenue to formulate a data structure is to subdivide the sample generation process into a series of subprocesses that appear in a consistent ordering for any given polymer active layer in an OFET, model the relationships among a standard domain of entities within those subprocesses, and use the resulting graph to sort data. Recently, Walsh et al. proposed a generalized polymer data structure in CRIPT, introducing a data format for process entities that can be sequentially arranged to represent successive material transformations.^29^ However, there is no established standard for defining the boundaries of a “process” for the sake of knowledge representation in materials data structures. We propose that incorporating a universal standard to help compose and arrange individual process stages would foster the adoption of generalized data models that can be used to model a broad set of application domains that are sensitive to complex processing histories.

**Figure 3:**
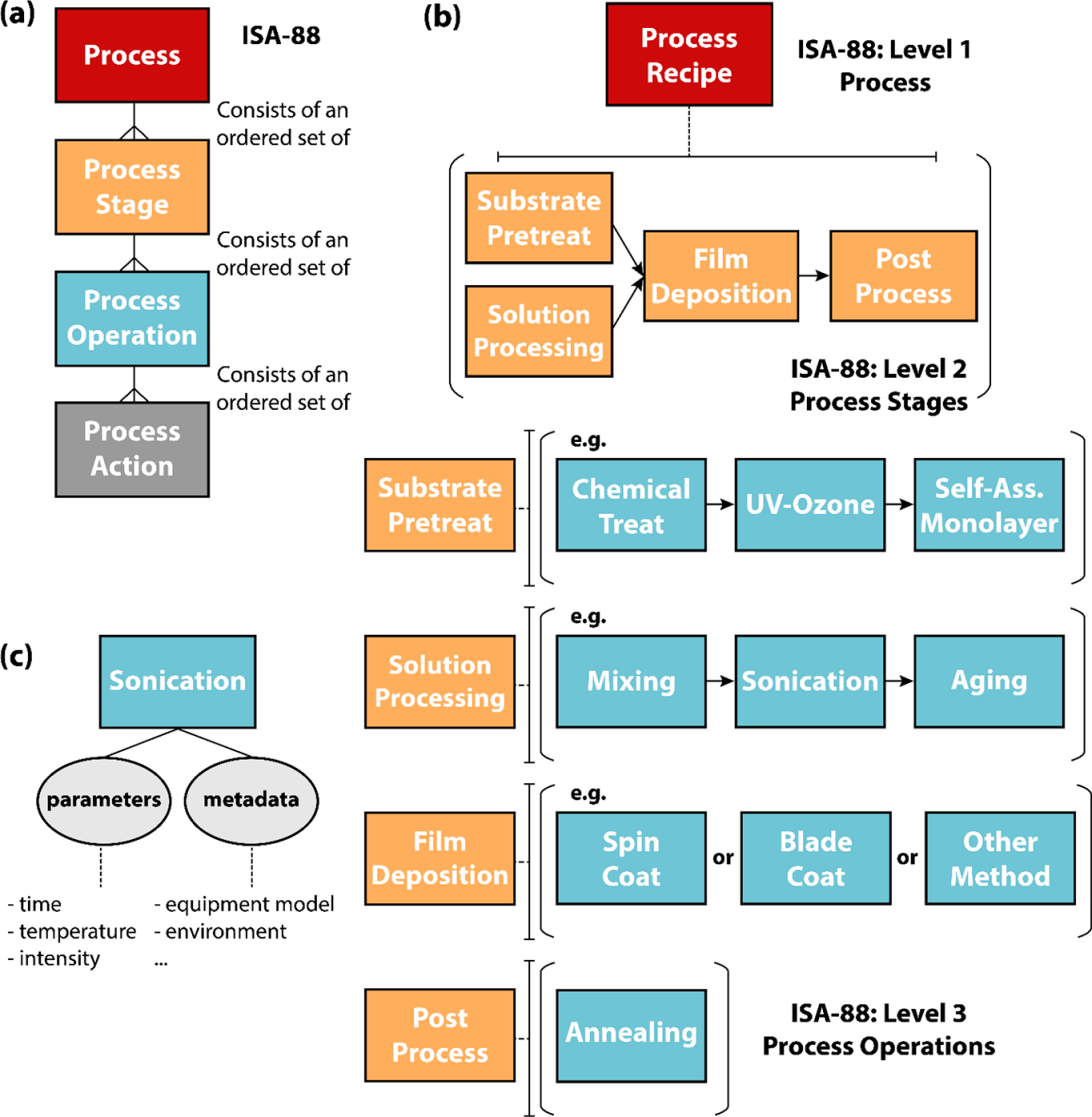
(a) Entity–relationship diagram of ISA-88, an international standard in batch process control. Material nodes and attributes are not explicitly included in the process model. Here, the former participate as inputs and outputs to the process, while attributes may describe a process node at the lowest level of complexity (see Figure 2). (b) Expanded graphical representation of an OFET fabrication recipe into a set of process stages using the second level of ISA-88. (c) Process stage expansion to directed relationships between process operations at the third level of ISA-88. Ovals represent attributes at the model’s lowest level.

Therefore, herein we adopt an international automation standard in ISA-88 to facilitate the conceptual modeling of the conjugated polymer deposition in a logical way (Figure 3a).^31^ ISA-88 is a standard that is used in batch process control to organize the various pieces of data associated with a complex network of instrumentation and process stages, wherein a batch process input material is fed to a defined order of processing actions (e.g., pieces of equipment) in series or in parallel to obtain some output material. Section 4.1 of ISA-88 defines a series of hierarchical subdivisions that are increasingly descriptive of an overall batch process. If the first level of the hierarchy is the overall process, the process is subdivided at the second level as an ordered set of process stages which operate independently from each other, usually in a planned sequence of physical changes in the material being processed. Process stages can be broken up into individual process operations, which are defined as major activities that result in a chemical or physical change in the material inputs. At the lowest level of the process model, process actions represent the minor activities that make up a process operation. Within each level of complexity, entities are a directed set of process subnodes organized in serial, parallel, or both.^31^

The conjugated polymer process recipe is expanded to process stages of substrate pretreatment, solution processing, film deposition, and postprocessing at the second level of ISA-88 (Figure 3b). These entities are *fixed stages* and represent logical subdivisions that broadly describe the active layer transformation of the input materials. Figure 3c represents a third level of process description, showing that the sequence of process operations that falls within the boundaries of each polymer process stage may vary from sample to sample. Substrate pretreatment refers to all of the processing activities related to preparing the patterned device substrate for the deposition process. This sequence may or may not include cleaning treatments (e.g., chemical washing, UV-ozone, plasma treatment, etc.) proceeded by one or more surface modification steps, such as the use of a self-assembled monolayer. Solution processing includes the various ordered steps (e.g., mixing, sonication, cooling, aging, etc.) that transform the polymer/solvent components into the final solution or ink formulation that ultimately gets coated onto the treated substrate during the film deposition stage. The film deposition stage includes only one node that contains information about the parameters and metadata of the coating method (e.g., blade coating, spin coating, drop casting, inkjet printing, etc.). Finally, postdeposition operations such as further chemical treatment or annealing appear under the postprocess stage node.

Further expanding the process operations into individual process actions is possible but provides a level of complexity that may not be required for sufficient database design, as the main purpose of the process model is to identify the most appropriate object classification to store attributes. For example, a “sonication” node might be expanded to process actions such as “set sonication time” or “set sonication intensity”, but this information can be just as easily represented by storing the attributes “sonication time” and “sonication intensity” in the parent process operation node (Figure 3c). However, it should be noted that this next level of process expansion may be useful for relating data commands or readings to computer-integrated or fully automated instrumentation. In either case, all of the raw parameters, data, and metadata information are stored in the nodes at the lowest process level.

## 3 OFET-db: A Database Implementation and Demonstration

The prior section proposes a general data structure and ontology for storing information related to the formation of the conjugated polymer active layer in a device such as an OFET. ISA-88’s sequenced process model also allows for process history to be effectively captured in a data model as the conjugated polymer is transformed into the final active layer of the measured device through a batch process. The process representation provides a high-level fixed structure (the main process stages of solution processing, substrate treatment, etc.) to promote aspects of a consistent relational schema while providing flexibility for storing dynamic information within each of the stages. Using the data model described above, an experimental repository of OFET device measurements was curated from a set of published, peer-reviewed literature data (Supporting Information) and unpublished laboratory data. The following section discusses the initial construction and continued curation of experimental device records into the database and provides a brief demonstration of utilizing the database for meaningful searching and data visualization.

### 3.1 Data Sourcing

The database was seeded using a set of experimental data sets for a set of three model polymer systems for electronic devices: P3HT, DPP-DTT, and poly([*N*,*N*′-bis(2-octyldodecyl)naphthalene-1,4,5,8-bis(dicarboximide)-2,6-diyl]-*alt*-5,5′-(2,2′-bithiophene)) (N2200). The P3HT, DPP-DTT, and N2200 data were curated from a body of literature combining over 50 peer-reviewed journal articles reporting OFET device performance containing a heterogeneous set of information including process-related parameters and some structural characterization data. A subset of the database was also curated using unpublished records from laboratory experiments.

### 3.2 Database Management System (DBMS)

The database was constructed using PostgreSQL, an open-source, relational database management system primarily based on the structured query language (SQL) that has strong support for NoSQL features. This allowed the database to have the preferred functionalities of the relational model (e.g., data normalization, data redundancy, error checking, etc.) while allowing for storage flexibility where attributes may be dynamic.^30^ Preserving relationships is also a key factor in representing sample provenance in a robust manner, which makes certain aspects of the relational model attractive for the sake of interoperability with other community databases. At the same time, PostgreSQL can handle storage and queries on a variety of complex data types, including JSON, XML, and binary objects, which is not a feature that is always available for SQL databases. The mix of SQL and NoSQL features allows the implementation of a data model that provides more convenient and robust organization for structured aspects (process stages, e.g., film deposition) and flexible storage for unstructured information or data fields that may evolve with research thrusts over time (process operations, e.g., coating parameters, and metadata fields). A complete description of the DBMS table schema, based on the data model described earlier, is available in the Supporting Information.

### 3.3 Vocabulary for Data Curation

The diversity of categorical or text descriptors in the OFET parameter space requires the use of a consistent vocabulary of keywords to guide the naming of attributes and free-text fields. Naming errors can in part be mitigated through built-in DBMS functionalities but may persist, especially in JSON formats, due to flexible key-value naming. At the time of writing, the implementation of OFET-db uses some keywords borrowed from other large-scale materials database efforts (e.g., where applicable and available, citation/source keywords from MaterialsMine,^26^ process and material keywords from CRIPT,^56^ etc.) but largely uses custom keywords that provide more specificity to descriptors relevant to the organic device fabrication domain (e.g., *blade*, *spin*, *inkjet*, *dip*, etc., to specify different classes of solution coating/deposition techniques). A full list of terms is available in the Supporting Information for the database implementation version described herein. Future design efforts will employ updated terminologies from shared community resources when available, as shared vocabularies promote consistent descriptions and interoperability. An experimental data entry template that incorporates this controlled vocabulary has also been adapted from a similar template shared by MaterialsMine.^27^ This template not only is intended to reduce the time and inconvenience that is inevitable for an experimentalist or domain expert to fill out a data record for database entry but provides a tool to reduce the error checking and validation workload on the back end. Future template versions could be implemented as user-friendly webforms, a web application, or directly coupled to electronic laboratory notebooks or integrated laboratory instrumentation to facilitate the process of database inserts for newly curated experimental records.

### 3.4 Data Visualization

Data reads from PostgreSQL are facilitated through built-in Python libraries, such as *psycopg2* and *pandas*. The following section demonstrates basic data analyses generated from such read queries to highlight the usefulness of enabling databases for experimental research purposes. In the future, it is envisioned that a larger population of data can facilitate data-driven knowledge discovery activities through the utilization of data science or machine learning techniques.

**Figure 4:**
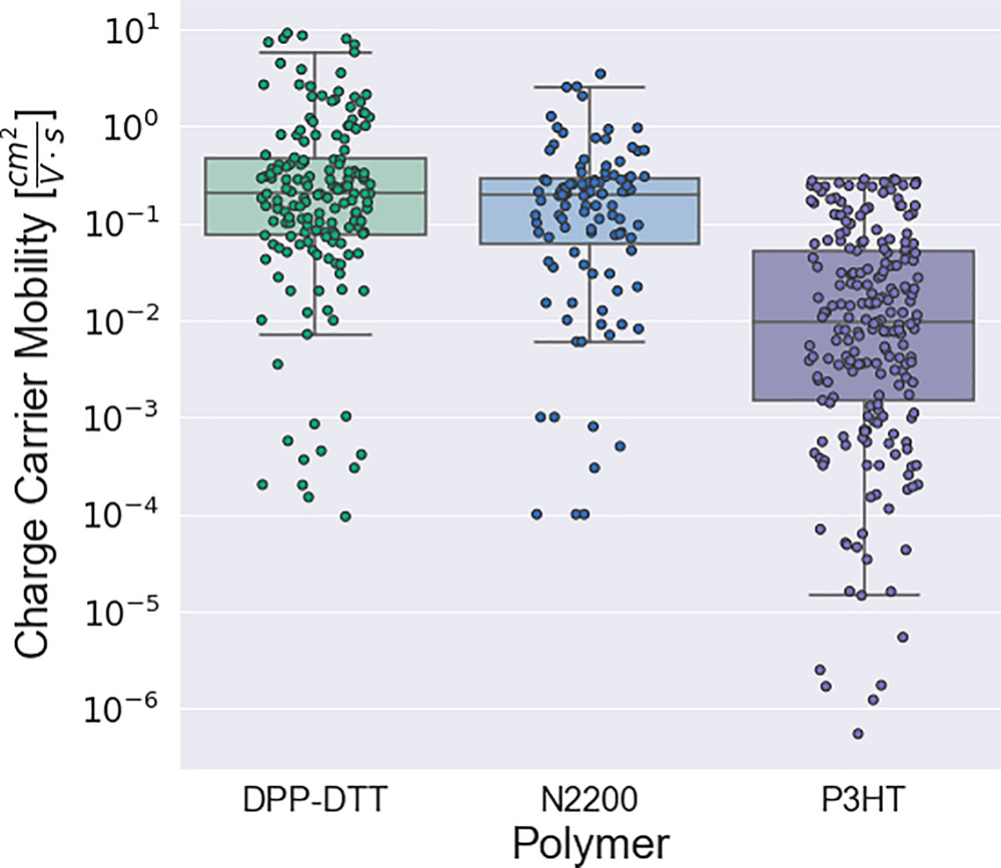
Single-axis scatterplots superimposed on boxplots, visualizing the mobility distributions for three representative polymers in OFET-db. The interquartile range (IQR) is defined between 25th and 75th percentiles, where the whisker end points are defined by 1.5 × IQR. Data is shown only for pure-component active layers; blends are omitted.

**Figure 5:**
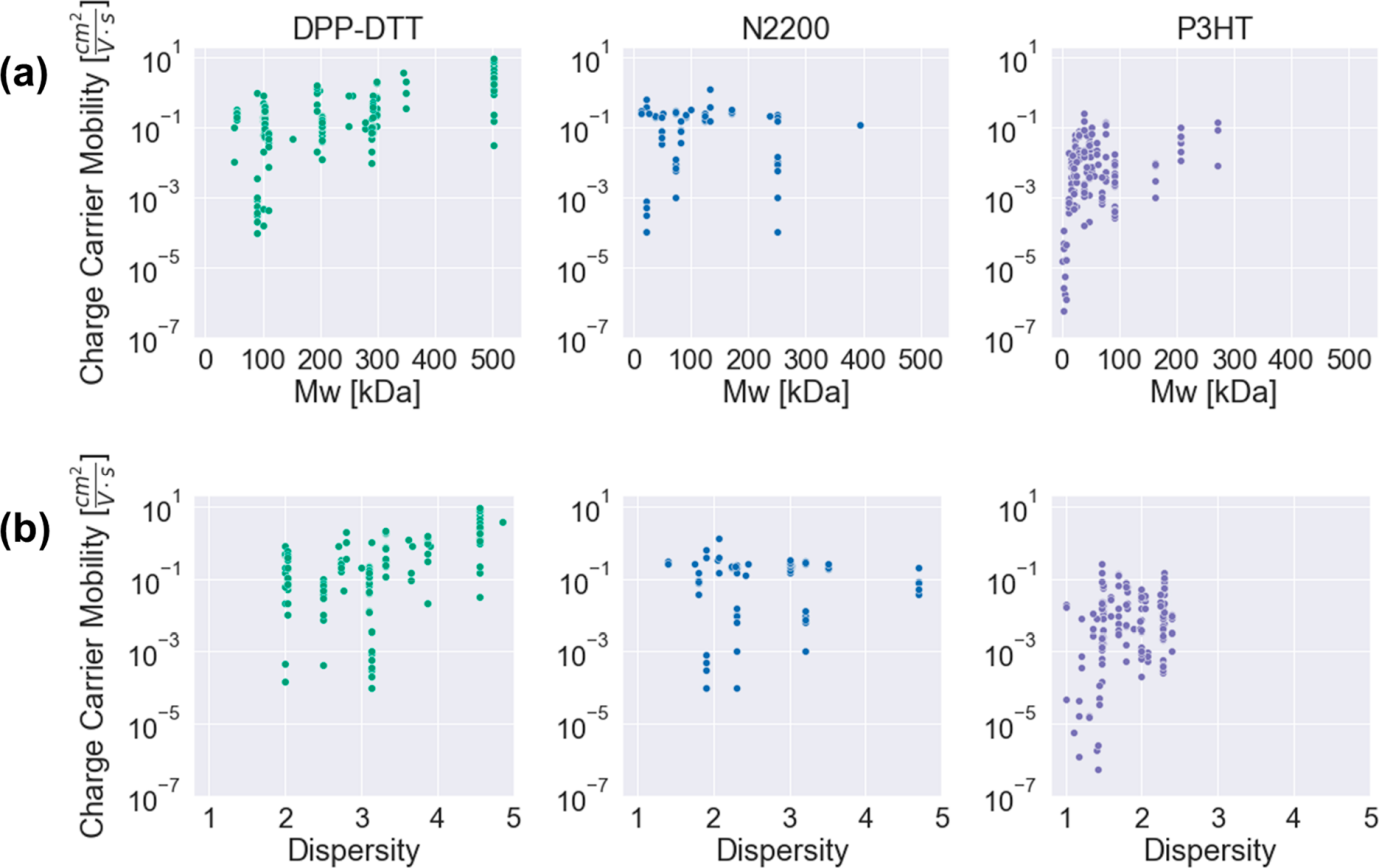
Charge-carrier mobility trends plotted against polymer material characteristics: (a) molecular weight and (b) dispersity.

Figure 4 shows the distribution of charge-carrier mobilities generated using data queried from OFET-db showing the total spread and statistics of performance values for three relatively well-represented polymers in OFET-db: DPP-DTT, N2200, and P3HT. OFET samples fabricated from all individual polymer types show performance variations that span several orders of magnitude. The higher average and maximum charge-carrier mobility achieved for the DPP-DTT and N2200 data also reflects the general performance advantage of donor–acceptor copolymers versus the model homopolymer P3HT, even despite this large variation. Polymer material characteristics, such as molecular weight and polydispersity, are important factors in performance differences, as demonstrated by Figure 5. Molecular weight is a well-studied parameter for conjugated polymers in OFETs, and it is generally understood that longer conjugated backbones promote entanglements and aggregates in solution and thereby enhance long-range molecular order and charge transport pathways in the thin film.^36,41,57^ The positive correlation between molecular weight and mobility is generally visible for P3HT and DPP-DTT. Variation in mobility for constant molecular weight is visible for all three polymers when, for example, the same study uses the same polymer batch to explore the effect of a chosen processing motif on the device performance, highlighting the importance of including such process details. A similar positive trend in mobility is visible for the polydispersity index (PDI), where devices made from a higher PDI polymer are more likely to have mobilities in the upper range of the data set.

Compared to molecular weight, however, the effect of PDI on the charge-carrier mobility is not as well understood by the community. While this behavior may at first glance be due to a correlation between molecular weight and PDI, a Spearman correlation analysis shows that these two variables are positively correlated only for P3HT (*r* = 0.79) but remain uncorrelated for DPP-DTT (0.03) and N2200 (0.23) (Table 1). PDI may be less frequently studied as a tunable experimental parameter since batch characteristics are sensitive to the synthetic procedure, hindering the design of controlled experiments for OFET performance comparisons. Recently, McBride et al. blended different *M*_w_ batches of P3HT and found that a wider molecular weight distribution exhibited beneficial effects due to a synergistic behavior between shorter tie chains connecting aggregated domains of larger chains in aged solutions.^58^ However, the data analysis above shows that a relationship between dispersity and mobility may be a common trend for copolymer systems, which potentially motivates a broader study into the structural mechanisms behind the mobility dependence on molecular weight distributions.

**Table 1:** Spearman Correlation Coefficients Calculated between Molecular Weight (*M*_w_) and PDI for Datasets Classified by Polymer

polymer	Spearman correlation coefficient: *M*_w_ vs PDI
DPP-DTT	0.03
N2200	0.23
P3HT	0.79

The data model implemented herein also provides the flexibility to index information from structural measurements (e.g., spectroscopic signals, microscopic images, etc.), which enhances the ability to interrogate process–structure–property relations. The population of structural measurements is much smaller than the number of device measurements, but here, we show that structural information is available and representative.

**Figure 6:**
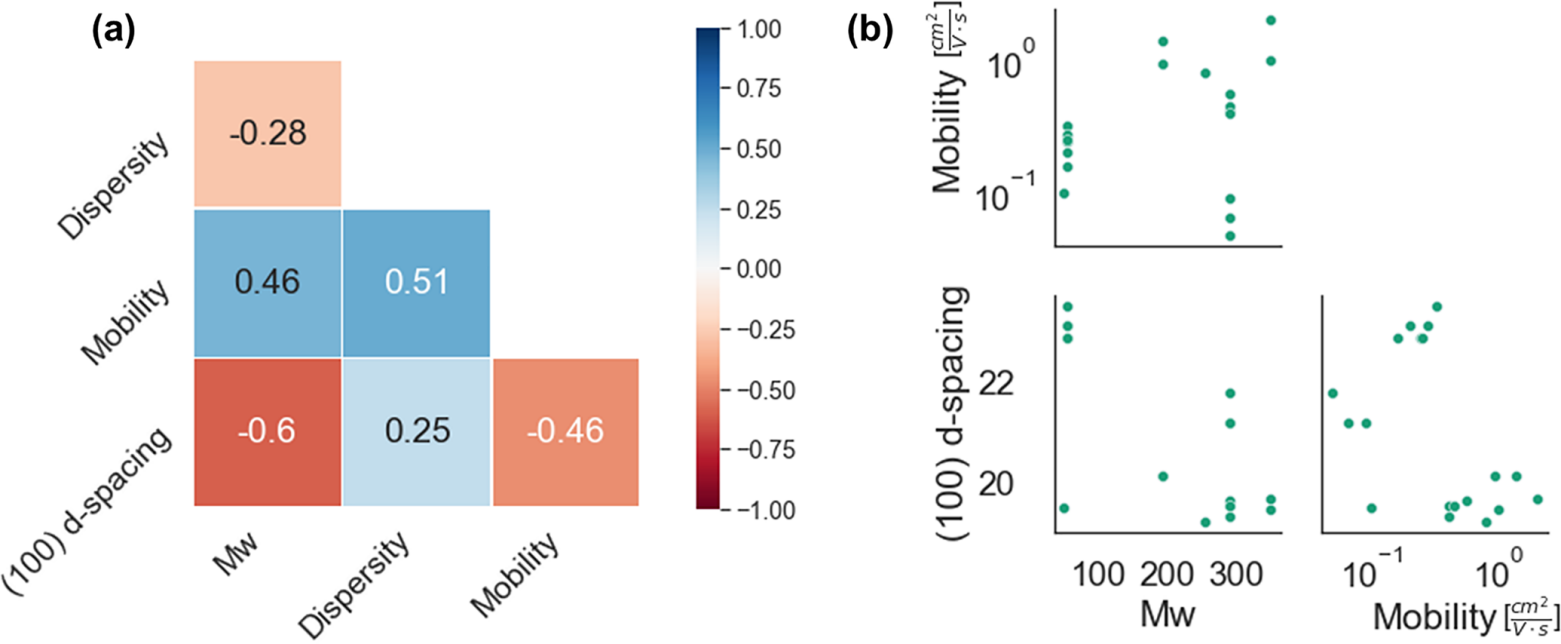
(a) Spearman correlation matrix on a subset of material information with thin film structural information ((100) *d* spacing) in GIWAXS. (b) Pair plots showing a 3-dimensional relationship between molecular weight, hole mobility, and available (100) *d* spacing from GIWAXS measurements of pure-component DPP-DTT devices.

For example, Figure 6 analyzes the polymer characteristics of DPP-DTT with respect to (100) *d* spacing extracted from available GIWAXS data. Most notably, Figure 6 shows a strong negative Spearman correlation value between molecular weight and (100) *d* spacing, indicating that the lamellar spacing tends to decrease with an increasing conjugated backbone length for DPP-DTT. A corresponding negative correlation between *d* spacing and mobility suggests that this change in lamellar spacing is a potential indicator for improved charge transport characteristics in the thin film, as decreased lamellar spacing could facilitate charge hopping.^59^ Though this distance is merely one factor characterizing the crystalline domain, this observation draws interest in considering further parameters (e.g., full width at half-maximum, degree of crystallinity, etc.) to study the impact on device performance. As the data reported here is relatively sparse, with only 31 of the DPP-DTT device samples registering associated GIWAXS data, more meaningful structure–property observations could potentially be extracted when a richer set of structural data is recorded. Nonetheless, we demonstrate the utility of using our preliminary body of populated data to inform future work toward a richer experimental repository and greater physical understanding. Such observations can suggest new hypotheses that can then be tested with additional experiments.

**Figure 7:**
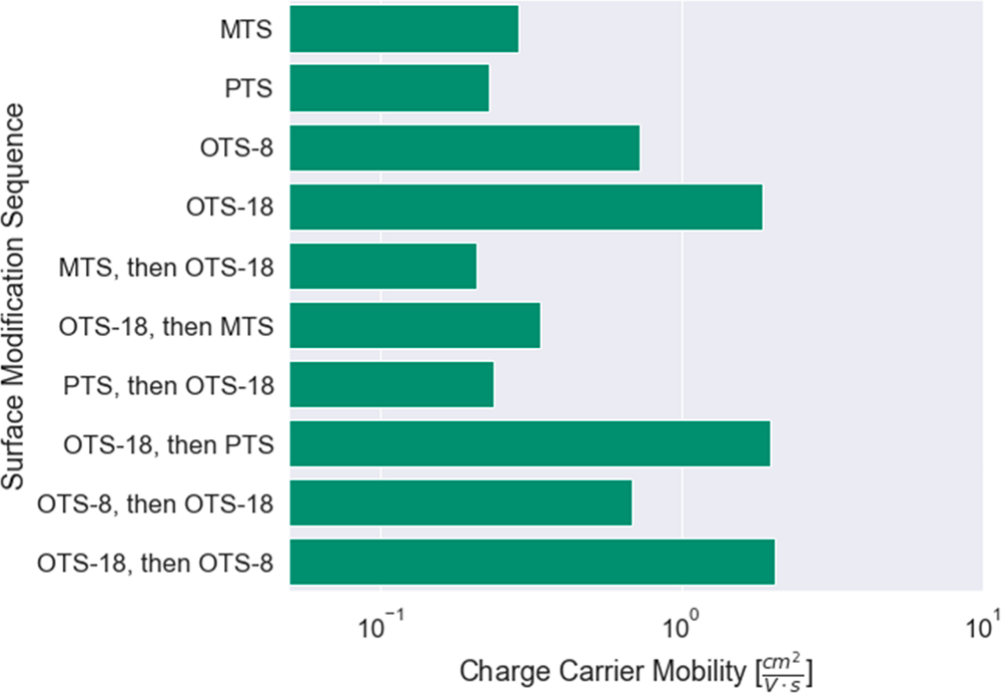
Impact of sequential substrate surface modification on charge-carrier mobility for a DPP-DTT study. Two different surface modifiers, including methyltrichlorosilane (MTS), octyltrichlorosilane (OTS-8), octadecyltrichlorosilane (OTS-18), and phenyltrichlorosilane (PTS), are used sequentially to treat the substrate as detailed in ref (38).

We expect that future data-driven studies that use OFET-db as a resource will benefit from representative storage of process history, as materials characteristics alone often provide insufficient information for fully understanding the experimental sensitivity of device performance. To that end, with our proposed process ontology we aim to enable the curation of experimental data with the associated process history of samples with the future intention of providing advanced analyses based on process recipes. A preliminary demonstration of the ability to curate, select, and plot process sequenced data is presented briefly herein. We used a query that searched for devices deposited on substrates treated by more than one surface modification agent (Supporting Information) to show the subset of data extracted from the database in Figure 7. This subset highlights results from a single study^38^ that compares the OFET mobility of devices deposited onto substrates sequentially pretreated with either of three pairs of silanes: methyltrichlorosilane (MTS) and octadecyltrichlorosilane (OTS-18), OTS-18 and phenyltrichlorosilane (PTS), and octyltrichlorosilane (OTS-8) and OTS-18. For all three of the pairings, noticeable differences in performance were observed depending on whether OTS-18 treatment was performed before or after the other silane agent.

This comparison highlights the necessity to capture process history (rather than simply process parameters) in fully describing device performance as such processes will affect the relationship between solution-state and thin-film assembly and therefore the final device properties. A major challenge, however, is that drawing device comparisons across different authors based on a standard recipe (i.e., isolating a large set of devices made with a standard set of process conditions that appear frequently) would require more data since the process space is very large.^60^ Currently, “low” availability of curated data precludes a more comprehensive meta-analysis of the process–structure–property relationships that govern the device performance of conjugated polymers. While the process–structure–property analyses discussed above for OFET-db demonstrate the challenge in driving data-driven studies in a “small data” environment, they also highlight the potential in mobilizing a database that can capture the various experimental nuances that could be indispensable toward greater physicochemical understanding of conjugated polymer-based organic devices. Therefore, the application of process ontologies is necessary for a broader adoption of representative materials databases, which the work herein addresses for conjugated polymer processing in OFETs.

## 4 Conclusions

Herein, we demonstrate the design and implementation of a data model for the experimental domain of OFETs as a foundation for broadly enabling database curation and management for organic thin-film electronics. Specifically, capturing process history in a manner that conforms to standard data protocols was a key challenge that was addressed by employing ISA-88, a batch process data model. Then, a database was constructed based on the model using PostgreSQL, enabling storage capabilities for both SQL (structured data) and NoSQL (document-based data) to provide flexibility without sacrificing the advantages of data redundancy/normalization in the relational model. While the work and discussion presented provides an experimental database that applies to the OFET active layer, it also serves as a model for adaptation to other aspects of organic device experiments by designing the data structure around an accepted process standard. Moving forward, enhancing materials ontologies to comprehensively capture classifications of process steps would facilitate the future design of data models in other domains that accurately manifest the real-world experimental processes. This is a necessary pursuit in elucidating the format in which a sample’s provenance is recorded within a database in FAIR data structures. Additionally, future work will build upon the preliminary body of curated experimental data to mobilize data-driven experimentation for polymer-based organic electronics.

## Data Availability

Supporting code and a local database implementation for OFET-db is available on GitHub at https://github.com/aaronliu64/ofetdb_public.

## Supporting Information Available

Detailed relational database table schema as implemented in PostgreSQL; vocabulary list for free-text attributes; list of publications from which initial seed data was extracted (PDF)
