# Supplementary material for: Conjugated Polymer Process Ontology and Experimental Data Repository for Organic Field-Effect Transistors
Source: Chem Mater. 2023 Oct 25;35(21):8816–26. doi: 10.1021/acs.chemmater.3c01842 (PMC10653076; doi:10.1021/acs.chemmater.3c01842)
Supplement: Supplementary file 1 — cm3c01842_si_001.pdf [file cm3c01842_si_001.pdf]

Supporting Information for:

Conjugated Polymer Process Ontology and Experimental Data Repository for Organic Field-Effect Transistors

Aaron L. Liu,[a] Myeongyeon Lee,[b] Rahul Venkatesh,[a] Jessica A. Bonsu,[a] Ron Volkovinsky,[a] J. Carson Meredith,[a] Elsa Reichmanis,[b*] Martha A. Grover[a*]

[a]School of Chemical & Biomolecular Engineering, Georgia Institute of Technology, 311 Ferst Drive, Atlanta, GA, 30332, USA

[b]Department of Chemical & Biomolecular Engineering, Lehigh University, Bethlehem, PA, 18015, USA

*Authors to whom correspondence should be addressed: elr420@lehigh.edu, martha.grover@chbe.gatech.edu

Includes:

Link to code and GitHub repository information for local copies of OFET-db
OFET-db table schema as implemented in PostgreSQL
Terminology and definitions of free-text fields implemented in OFET-db
List of publications from which initial seed data was extracted for OFET-db

S1. DATABASE

Please see https://github.com/aaronliu64/ofetdb_public for instructions and files required for running a local server of OFET-db, as well as updates on new public releases.

S2. SCHEMA DESIGN

The main manuscript proposes a general data model for storing information related to the process transformation of the conjugated polymer to the active layer in an OFET. To demonstrate how the data model is translated from the conceptual schema to a relational schema, the following information details technical and organizational considerations in constructing a repository of experimental records for OFETs.

High-level nodes

The high-level entity-relationship diagram in Figure 2 shows a network between **sample**, **experiment**, **measurement**, and **process recipe** objects. Figure S1 provides a visual representation that illustrates these linkages through a table column structure. Data redundancy is mitigated by separating information into tables based on the conceptual design; foreign keys within each table denote linkages that reference information defined by relationships to other tables. In a relationship between two entities, the edges along with their cardinality (i.e., 1 to 1, 1 to N) provide guidance on which table should contain the foreign key that references the other table. The **sample** table participates in a many-to-one relationship with **experiment**, as one experimental source may report multiple sample records, but a given sample may only belong to one experiment. It then follows that the foreign key *exp_id* should be stored in the **sample** table, which has the cardinality of 1 (*i.e.*, in 1 to N). Similar semantics are applied for the one-to-many relationships that exist along the other edges (*i.e.*, a sample could have many measurements, but a single measurement

must refer to only one sample; many samples could be made with the same process recipe, but a single sample is only made from one recipe).

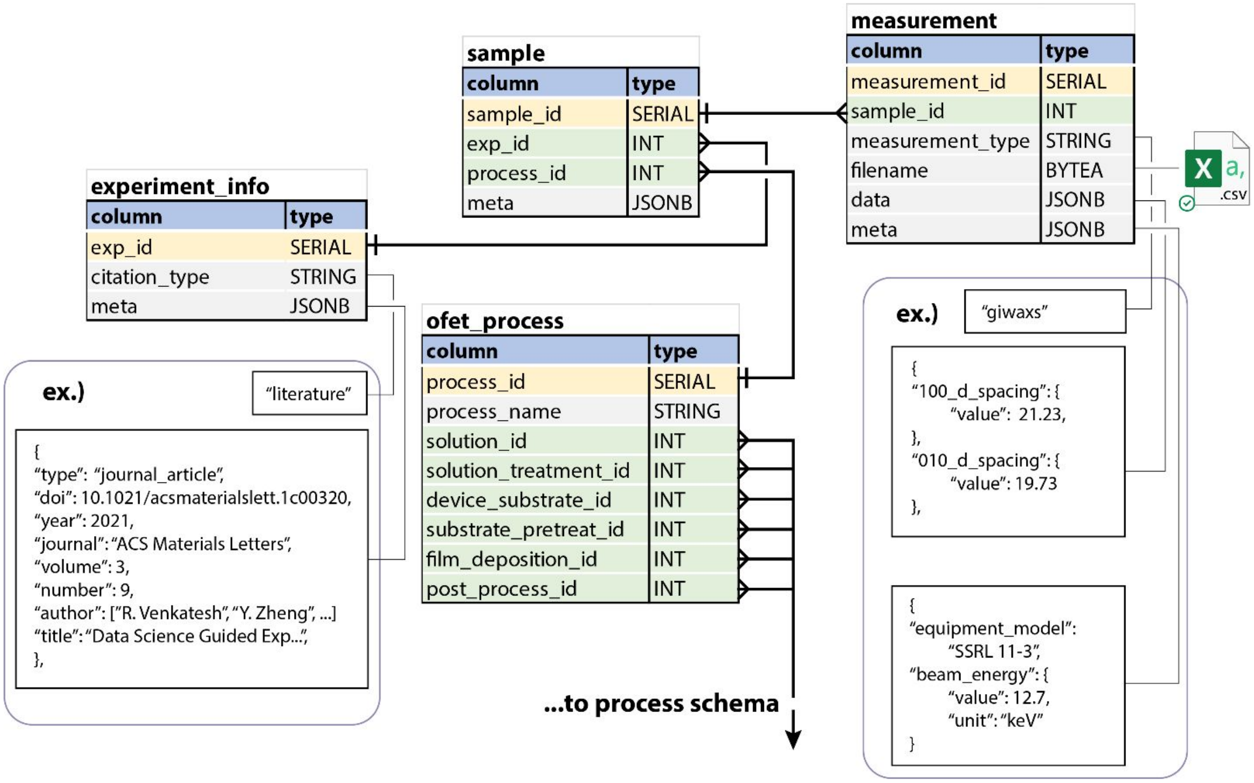

Figure S1. The table schema implemented for the relational DBMS in PostgreSQL, adapted from the entity-relationship diagram that is displayed in Figure 2

Javascript Object Notation (JSON) document formats (*i.e.*, documents that store key-values pairs which need not conform to the same schema) are utilized wherever flexibility is required to represent the information in an attribute. Some database management systems (DBMSs) support JSON or JSON-like datatypes, which can be leveraged to implement attributes such as *data*, *meta*, or *params* which may have a different format if the table has a *type* attribute. For example, a **measurement** of type *transfer_curve* may include attributes such as "hole_mobility", while a structural measurement of type *giwaxs* may include attributes such as *100_dspacing*.

Material nodes

Reducing data redundancy is especially important in input material nodes, as polymers, solvents, and device substrate designs will frequently be re-used across different experimental studies (Figure S2). The **device_substrate** and **solution** tables are referenced by the parent **process recipe** node, as they participate as material inputs to the device fabrication process. The **device_substrate** table includes the parameters and metadata associated with the OFET device geometry, electrode configuration, and gate/dielectric layers. Preventing data redundancy of materials species requires the use of unique chemical identifiers. The storage of solvent information, for example, is accomplished by indexing the PubChem CID, which is a universal identifier that works for distinguishing most small molecules. PubChem also provides an API that can easily provide links to other chemical identifiers, such as SMILES, InChI, and others, and is generally an acceptable method for FAIR chemical representation. Polymers are more challenging because their stochasticity is a roadblock to providing unique identifiers. A unique polymer record contains information that is specific to the synthesis history of the polymer, which may not always be available. The current implementation of the **Polymer** node includes batch properties such as the polymer name, basic molecular weight information (M_n, M_w, dispersity, tacticity, *etc.*), manufacturer, batch number, etc. about from where the material is derived, which is generally a feasible solution when only considering a small number of monomer types. Providing unique, FAIR identifiers and characterization data structures for polymer names is an active effort in the polymer informatics community (*i.e.*, BigSMILES,[1] ChemProps[2]), and will be a major design consideration in future database scaleup.

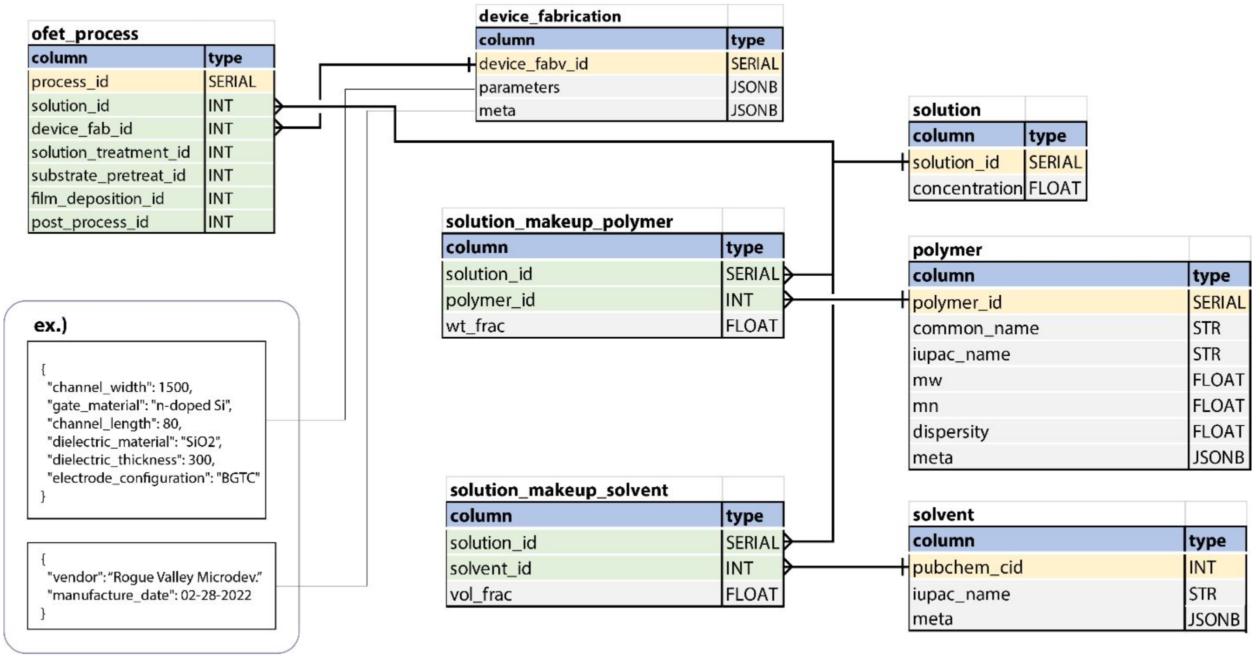

Figure S2. Table schema for materials nodes, including a JSON schema example for device substrate information. To deal with the many-to-many relationships in the Solution entity, polymer and solvent components are respectively linked to the solution through a component table (solvent_makeup and polymer_makeup) which contains information about component blend ratios for polymer and solvent species.

A solution is a multicomponent mixture of polymer and solvent components, which produces a many-to-many relationship in the data model. One possible way to represent this information in the solution table is by storing an array of polymer identifiers and an array of solvent identifiers with their respective weight/volume fractions in solution. However, storing these arrays creates challenges with proper data referencing, as often the DBMS cannot search through arrays to ensure identifiers are updated. Achieving data normalization in this scenario is accomplished by providing component linking tables for the polymer-solution (*polymer_makeup*) and solvent-solution (*solvent_makeup*) relationships respectively. For example, in the polymer case, *polymer* contains information about the material used, *solution* represents the solution used in the experiment, and the table *polymer_makeup* references both the *polymer* and *solution* tables and links them with a weight fraction attribute.

Process nodes

The two major considerations in designing tables for the process nodes is implementing flexibility for different types of sub-processes and representing the sequence order of those sub-processes. Figure S3 shows the relational table schema for the process nodes. Similar to other portions of the schema, sub-process flexibility is implemented by allowing a *treatment_type* attribute, and flexible JSON types that contain the parameters specific to that class of treatments. The **film_deposition** table may include records with different types of coating processes, where parameters *spin_rate* or *spin_time* may only apply to a *deposition_type* = *"spin_coat"* and parameters *blade_speed* and *stage_temperature* only apply to *deposition_type* = *"blade_coat"*. For the other tables in which order matters, individual instances of sub-processes are treated as recipe steps that can be reused (Figure S3, right).

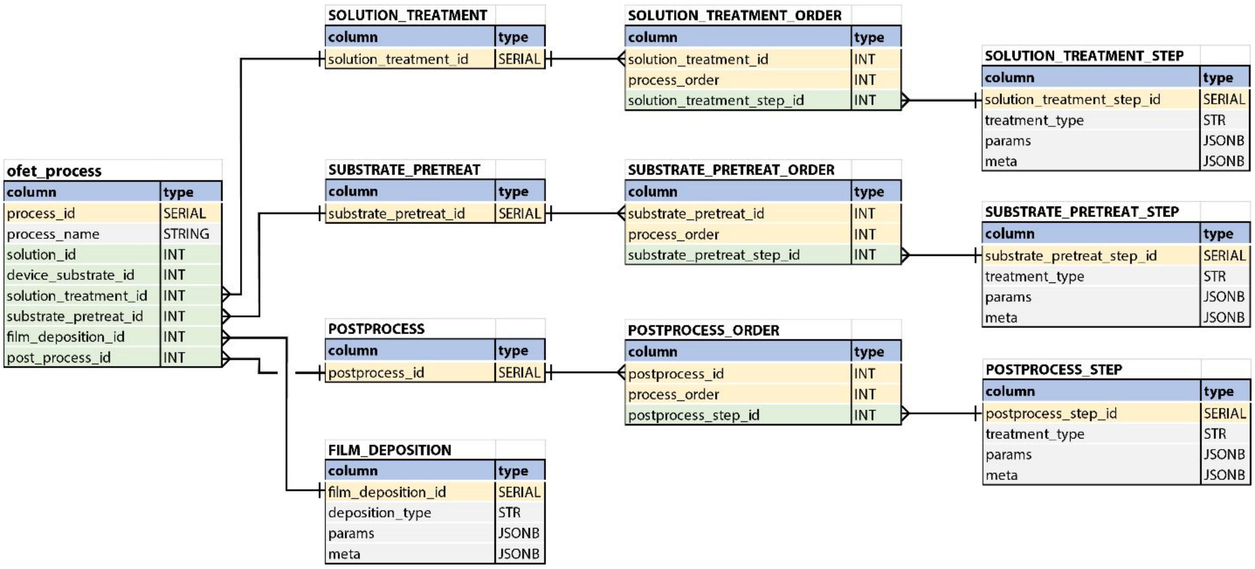

Figure S3. Table schema for process nodes, expanded down to process operations

To account for process order, we can imagine a scenario similar to the many-to-many **solution** mixture, where an initial solution for storing a particular instance of a **solution_treatment** recipe might just store an ordered array of indexes that correspond to

treatment step information. Similarly, storing arrays of references leads to data redundancy issues, so each process operation is data normalized by providing a table that takes an overall sub-process recipe (*i.e.,* **solution_treatment**) and its component recipe steps (*i.e.,* **solution_treatment_step**), and subsequently links them through an ordering table (*i.e.,* **solution_treatment_order**) that transforms process parameters into process history.

Table S1. Example controlled vocabulary defined for OFET-db implementation in PostgreSQL. This table lists the main attributes attached to the parent relations, and a description of each

parent	name	description
experiment_info	citation_type	Data is extracted from peer-reviewed literature, or unpublished from lab data (i.e., local lab notebooks)
experiment_info	first_name	Data contributor's name (ex. 'Rahul')
experiment_info	last_name	Data contributor's name (ex. 'Venkatesh')
experiment_info	email	Data contributor's institutional email (ex. 'rvenkatesh6@gatech.edu')
experiment_info	sample_date	mm/dd/yyyy that the OFET sample was fabricated
experiment_info	lab_notebook_id	Lab notebook number and page: ex.) RV_1.99
experiment_info	lab_sample_id	sample identifier in the lab notebook: ex.) M317_5gL
experiment_info	doi	Digital object identifier, for peer-reviewed literature
solution	concentration	The total concentration of all solute species in the solution
solvent	pubchem_cid	PubChem CID: https://pubchem.ncbi.nlm.nih.gov/
solvent	iupac_name	Preferred name from IUPAC. Listed under "IUPAC Name" in PubChem. Note that CRIPT vocab has "preferred_name"
solvent	boiling_point	Boiling point of the solvent
solution_makeup_solvent	vol_frac	Volume fraction of the solvent, relative to total volume of solvent species
polymer	iupac_name	ex.) poly[2,5-(2-octyldodecyl)-3,6-diketopyrrolopyrrole-alt-5,5-(2,5-di(thien-2-yl)thieno [3,2-b]thiophene)]
polymer	common_name	DPP-DTT
polymer	mn	Molecular weight, number average
polymer	mw	Molecular weight, weight average
polymer	dispersity	Dispersity index, or polydispersity. Calculated also with Mw/Mn
polymer	supplier	If not synthesized in-house, the company name of the supplier. Ex.) Rieke Metals
polymer	batch_number	Batch identifier number, if vendor provided
polymer	regioregularity	Regioregularity of polymer, such as for P3HT
polymer	sidechain	side chain chemical formula (if applicable)
solution_makeup_polymer	wt_frac	Mass fraction of the polymer, relative to total volume of polymer species
(multiple sub-processes)	treatment_type	Keyword description of the process step
(multiple sub-processes)	environment	The ambient environment in which the process occurred
(multiple sub-processes)	mixing_speed	Parameter setting for agitation
(multiple sub-processes)	temperature	Temperature setting for process

(multiple sub-processes)	time	Length of time for process
(multiple sub-processes)	intensity	Power output (e.g., in sonication, irradiation)
(multiple sub-processes)	wavelength	Wavelength for light output
(multiple sub-processes)	frequency	Frequency of a perturbation, such as vibration or sonication
solution_treatment	pubchem_cid	PubChem identifier for chemical used in process step
solution_treatment	vol_frac_added	Amount of solvent added relative to total volume of the solution
solution_treatment	equipment_model	Model information for equipment used
device_fabrication	electrode_configuration	Arrangement of the gate and contact electrodes
device_fabrication	channel_length	Characteristic distance between channel source and drain features (Perpendicular to charge carrier direction)
device_fabrication	channel_width	Feature size perpendicular to charge carrier direction
device_fabrication	adhesion_layer	Adhesion layer deposited to facilitate contact adhesion
device_fabrication	electrode_material	Conducting material used for the contacts
device_fabrication	gate_material	Material that makes up the gate contact
device_fabrication	dielectric_material	Material that makes up the dielectric layer
device_fabrication	dielectric_thickness	Thickness of the dielectric layer
device_fabrication	dielectric_capacitance	Capacitance of dielectric layer
film_deposition	deposition_type	Keyword description of deposition step
film_deposition	spin_rate	Coating speed (spin coating only)
film_deposition	spin_time	Coating time (spin coating only)
film_deposition	injection_method	Spin coating process step, if solution was deposited while static or while spinning
film_deposition	coating_speed	Coating speed (all methods except spin coating)
film_deposition	blade_angle	Angle of coating blade with respect to substrate
film_deposition	blade_material	Material of coating blade
film_deposition	blade_height	Height of coating blade
film_deposition	vibration_frequency	Frequency of vibration for assisted blade coating
film_deposition	vibration_amplitude	Amplitude for assisted blade coating
film_deposition	coating_direction	Coating direction (i.e., for meniscus-guided techniques) relatiive to the channel direction
film_deposition	deposited_volume	Amount of solution deposited for coating

Table S2. Representative process labels associated with process operation types, and a description of each

**process descriptor**	**name**	**description**
treatment_type	mixing	mixing

treatment_type	poor_solvent	addition of poor solvent a liquid mixture
treatment_type	uv_irradiation	exposure of solution to uv light
treatment_type	aging	static time exposure to induce physical/chemical change
treatment_type	sonication	sonication
treatment_type	chemical_treat	some exposure in the presence of a chemical
treatment_type	uv_ozone	UV ozone cleaning, i.e., of a surface
treatment_type	sam	self-assembled monolayer
treatment_type	annealing	heat treatment often with slow, controlled cooling process
deposition_type	spin	spin coating
deposition_type	blade	blade coating, using a doctor blade or mounted plate
deposition_type	drop	drop casting
deposition_type	inkjet	inkjet printing
deposition_type	wire	wire-bar coating
deposition_type	dip	dip coating
deposition_type	spray	spray coating

Table S3. Representative measurement labels associated with flexible document storage attributes, and a description of each

**measurement descriptor**	**name**	**description**
measurement_type	hole_transfer	hole charge carrier transfer behavior
measurement_type	electron_transfer	electron charge carrier transfer behavior
measurement_type	uv_vis_film	UV-vis spectrum, on a film
measurement_type	uv_vis_solution	UV-vis spectrum, on solution
measurement_type	giwaxs	Grazing Incidence Wide-Angle X-ray Scattering
measurement_type	afm_height	atomic force microscope height map
measurement_type	afm_phase	atomic force microscope phase map
measurement_type	thickness	film thickness
measurement_type	viscosity	viscosity
error	stderr	standard error
error	stdev	standard deviation
error	ci_95	95% confidence interval
device	measurement_type	Keyword description of measurement type
device	direction	Transfer curve direction
device	mobility	Charge carrier mobility
device	mobility_regime	Transfer curve regime in which mobility was calculated
device	threshold_voltage	Minimum gate-to-source voltage needed to turn on a transistor
device	on_off_current	Maximum over minimum current at constant drain voltage

device	subthreshold_voltage	
device	measurement_environment	Ambient environment in which OFET was measured
device	measurement_temperature	Temperature at which OFET was measured (such as on a controlled stage, etc.)
device	Vds	
device	Vg_range	Transfer curve parameter range for Vg
device	filepath	File name of the attached measurement file (including extension, i.e., 'RV1.66_4_ch1.csv'

Table S4. List of publications containing OFET mobility data used to initially populate the demonstrated OFET-db.

doi	First Author	Year	Journal
10.1039/C5TC02579F	Y. Lei	2015	Journal of Materials Chemistry C
10.1021/acs.chemmater.7b03019	G. Zhang	2017	Chemistry of Materials
10.1021/acs.chemmater.8b05224	G. Zhang	2019	Chemistry of Materials
10.1038/srep00754	J. Li	2012	Scientific Reports
10.1038/srep24476	Y. Lei	2016	Scientific Reports
10.1016/j.cplett.2020.137507	T. Afzal	2020	Chemical Physics Letters
10.1021/acs.chemmater.0c02199	T. Sarkar	2020	Chemistry of Materials
10.1016/j.spmi.2018.12.022	M. J. Iqbal	2019	Superlattices and Microstructures
10.1039/c8sm02517g	Y. Xi	2019	Soft Matter
10.1002/adfm.201909787	P. Kafle	2020	Advanced Functional Materials
10.1063/1.5135977	H. Park	2020	APL Materials
10.1063/1.5135977	H. Park	2020	APL Materials
10.1039/c5tc02133b	A. Armin	2015	Journal of Materials Chemistry C
10.1039/c3nr03989g	H. Xu	2013	Nanoscale
10.1021/acsami.8b03288	S.W. Baek	2018	ACS Applied Materials & Interfaces
10.1021/acsami.7b08133	G. Qu	2017	ACS Applied Materials & Interfaces
10.1021/acsami.7b08133	G. Qu	2017	ACS Applied Materials & Interfaces
10.1021/acsmaterialslett.1c00320	R. Venkatesh	2021	ACS Materials Letters
10.1126/sciadv.1600076	H. Luo	2016	Science Advances
10.1002/adma.201102786	Z. Chen	2011	Advanced Materials
10.1038/ncomms16070	E. Mohammadi	2017	Nature Communications
10.1002/adfm.201706372	Y. Lei	2018	Advanced Functional Materials
10.1021/am507759u	Y. Karpov	2015	ACS Applied Materials & Interfaces
10.1002/adfm.201604744	M.M. Nahid	2017	Advanced Functional Materials
10.1002/aelm.201700559	M.M. Nahid	2018	Advanced Electronic Materials
10.1021/acsami.0c07385	Y. Kim	2020	ACS Applied Materials & Interfaces
10.1021/acs.chemmater.8b05114	J. Choi	2019	Chemistry of Materials
10.1073/pnas.1501381112	S. Wang	2015	Proc. of the Nat. Academy of Sciences

10.1002/aelm.202101324	T. Steckmann	2022	Advanced Electronic Materials
10.1021/acs.macromol.1c01661	L. Tang	2021	Macromolecules
10.1021/acs.chemmater.5b03775	N. Kim	2015	Chemistry of Materials
10.1063/1.3424792	M. Caironi	2010	Applied Physics Letters
10.1038/srep03425	A. Luzio	2013	Scientific Reports
10.1039/C4TC00390J	G. C. Schmidt	2014	J. Mater. Chem. C
10.1021/acsapm.2c00554	L. Tang	2022	ACS Applied Polymer Materials
10.1021/acs.macromol.1c02329	S. Wang	2022	Macromolecules
10.1038/s41598-020-60812-x	S. Brixi	2020	Scientific Reports
10.1002/adfm.201807786	Y. Kim	2019	Advanced Functional Materials
10.1002/adfm.201002729	A. R. Aiyar	2011	Advanced Functional Materials
10.1021/am3027822	A. R. Aiyar	2013	ACS Applied Materials Interface
10.1016/j.orgel.2011.06.027	U. Bielecka	2011	Organic Electronics
10.1021/cm049617w	J. Chang	2004	Chemistry of Materials
10.1103/PhysRevB.74.115318	J. Chang	2006	Physical Review B
10.1021/nn401323f	M. Chang	2013	ACS Nano
10.1063/1.2400796	S. Cho	2006	Journal of Applied Physics
10.1002/adfm.201403708	D. Choi	2014	Advanced Functional Materials
10.1007/s11801-011-0122-z	C. Jiang	2011	Optoelectronics Letters
10.1002/adma.200305275	R.J. Kline	2003	Advanced Materials
10.1021/ma047415f	R.J. Kline	2005	Macromolecules
10.1021/ma2000515	G. M. Newbloom	2011	Macromolecules
10.5012/bkcs.2014.35.8.2277	Y. D. Park	2014	Bulletin of the Korean Chemical Society
10.1021/jp4116047	B. Park	2014	The Journal of Physical Chemistry C
10.1002/polb.23022	C. Scharsich	2011	Polymer Physics
10.1063/1.2222065	M. Surin	2006	Journal of Applied Physics
10.1016/j.synthmet.2006.04.013	H. Fu	2006	Synthetic Metals
10.1002/adfm.200400017	A. Zen	2004	Advanced Functional Materials
10.1002/adfm.201301007	K. Zhao	2013	Advanced Functional Materials

References

(1) Lin, T.-S.; Coley, C. W.; Mochigase, H.; Beech, H. K.; Wang, W.; Wang, Z.; Woods, E.; Craig, S. L.; Johnson, J. A.; Kalow, J. A.; et al. BigSMILES: A Structurally-Based Line Notation for Describing Macromolecules. *ACS Central Science* **2019**, *5* (9), 1523-1531. DOI: 10.1021/acscentsci.9b00476.

(2) Hu, B.; Lin, A.; Brinson, L. C. ChemProps: A RESTful API enabled database for composite polymer name standardization. *Journal of Cheminformatics* **2021**, *13* (1), 22. DOI: 10.1186/s13321-021-00502-6.
